# Nanostructured Metal Oxide Gas Sensors, a Survey of Applications Carried out at SENSOR Lab, Brescia (Italy) in the Security and Food Quality Fields

**DOI:** 10.3390/s121217023

**Published:** 2012-12-12

**Authors:** Andrea Ponzoni, Elisabetta Comini, Isabella Concina, Matteo Ferroni, Matteo Falasconi, Emanuela Gobbi, Veronica Sberveglieri, Giorgio Sberveglieri

**Affiliations:** 1 SENSOR, CNR-IDASC, UOS Brescia, Via Branze 45, 25123 Brescia, Italy; 2 SENSOR, Dipartimento di Ingegneria dell’Informazione, Università degli Studi di Brescia, Via Valotti 9, 25133 Brescia, Italy; E-Mails: elisabetta.comini@ing.unibs.it (E.C.); isabella.concina@ing.unibs.it (I.C.); matteo.ferroni@ing.unibs.it (M.F.); matteo.falasconi@ing.unibs.it (M.F.); giorgio.sberveglieri@ing.unibs.it (G.S.); 3 DISA, Università di Udine, Via Scienze 208, 33100 Udine, Italy; E-Mail: micol@uniud.it; 4 Dipartimento di Scienze Agrarie e degli Alimenti, Università di Modena e Reggio Emilia, Via Amendola 2, 42100 Reggio Emilia, Italy; E-Mail: veronica.sberveglieri@unimore.it

**Keywords:** metal oxides, chemisresistors, electronic nose, nanowires, thin films, DMMP, chemical warfare agents, multivariate analysis, microbial spoilage, *Alicyclobacillus*

## Abstract

In this work we report on metal oxide (MOX) based gas sensors, presenting the work done at the SENSOR laboratory of the CNR-IDASC and University of Brescia, Italy since the 80s up to the latest results achieved in recent times. In particular we report the strategies followed at SENSOR during these 30 years to increase the performance of MOX sensors through the development of different preparation techniques, from Rheotaxial Growth Thermal Oxidation (RGTO) to nanowire technology to address sensitivity and stability, and the development of electronic nose systems and pattern recognition techniques to address selectivity. We will show the obtained achievement in the context of selected applications such as safety and security and food quality control.

## 1. Introduction

Conductometric gas sensors based on semiconducting metal oxides are among the most promising solid state gas sensors thanks to their high sensitivity to a broad range of chemicals, reduced size and weight, low power consumption, compatibility with silicon technology, possibility to produce these devices by means of cheap techniques compatible with industrial scaling up such as sputtering or evaporation and condensation methods.

Since the first paper from Brattain *et al.*[1], reporting in the 50s that the electrical conductance of semiconductors could be modulated by gas adsorption/desorption phenomena, several groups all over the World have been dedicated to study this principle to develop gas sensors.

In this Special Issue dedicated to the *State of the art of Sensors in Italy*, we report the recent achievements obtained at the SENSOR Lab in Brescia. To properly frame these results, the paper starts with a short introduction on the working principle of metal oxide chemiresistors and reports the strategies followed at SENSOR to optimize the structure of sensitive layer, from thin film to nanowire technology. The gas-sensing performances of these technologies are compared choosing the detection of chemical warfare agents (CWAs) as target application. Finally the results obtained integrating these devices in artificial olfactory systems (AOSs) are shown focusing on three paradigmatic applications of food-quality control, namely detection of bacteria-contamination in beverages, identification of fraud in extra virgin olive oil and identification of errors in a tomato processing line.

## 2. Working Principle

Conductometric gas sensors, also named chemiresistors, transduce the presence in the atmosphere of a given chemical compound through a variation of their electrical resistance. They are based on semiconducting metal oxides, whose electrical properties are modulated by red-ox interactions with adsorbing gaseous molecules.

In particular, active species, such as O^−^, O_2_^−^, O^2−^, OH^−^, have been identified as the active centers responsible for the above red-ox reactions [2]. Such species cover the oxide surface with their relative population depending on the oxide temperature and atmospheric composition [3].

In the typical temperature range of metal oxide chemiresistors (200–500 °C), O_2_^−^ ions are the most abundant at low temperature (below 300–350 °C), while a higher temperature favors the dissociation of molecular oxygen leading to atomic oxygen ions O^−^.

From an electrical point of view, when a semiconducting oxide is exposed to air, the adsorption of water and/or oxygen from the atmosphere modifies the band structure of the material at the surface with respect to the bulk. Chemisorption, involving the transfer of electrons between the conduction of the semiconductor and the adsorbed atom/molecule, is that particular form of adsorption responsible for building up the population of active ions at the oxide surface and the consequent band bending [4]. In particular, chemisorption of oxygen creates acceptor surface states that withdraw electrons from the outermost layer of the semiconductor, thus inducing a surface up-ward bending of the oxide band structure. In the abrupt approximation, such band bending is described by a surface potential V_S_, and a space charge layer (SCL) of width W, which are determined by morphological and electronic parameters according to Equations (1) and (2), respectively [4]:

(1) $$V_{s}=\frac{{\mathrm{qN}}_{s}^{2}}{{2ɛN}_{B}}$$

(2) $$W=\sqrt{\frac{{2ɛV}_{s}}{{\mathrm{qN}}_{B}}}$$

Here, q is the elementary charge, ε is the electrical permittivity of the material, N_B_ is the density of charge carriers in the bulk, N_S_ is the density of ionized surface states. So far, the value of W depends both on the properties of the material (ε and N_B_) as well as on the amount of adsorbed species that creates surface states and thus a surface barrier. Typical values ranges from a few nm to a few tens of nm [4].

In n-type semiconductors, such as SnO_2_, WO_3_ or ZnO, such an upward band bending will lead to a conductance decrease, while it will decrease the conductance of p-type semiconductors, such as CuO or NiO.

The further interaction of gaseous molecules with the aforementioned active ions modulates their population over the semiconductor surface and thus the electrical properties of the material. According to this mechanism, reducing gases, such as CO and hydrocarbons, get oxidized reacting with oxygen ions and their population over the oxide surface decreases, thus increasing the material conductance (for n-type semiconductors). Oxidizing gases, such as NO_2_ and O_3_, are reduced by the interaction with the oxide surface and, as a consequence, the population of oxygen ions increases as does the material resistance (for n-type semiconductors).

Despite their qualitative nature, these arguments are effective to explain the basic ideas underlying the strategies adopted to design and optimize metal oxide layers for conductometric gas sensors. Several approaches have been adopted to develop highly performing metal oxide layers, which can be grouped into two ideal structures, named as thin-film and thick-film, according to the main preparation techniques typically adopted to prepare layers with these structures.

**Figure 1.**
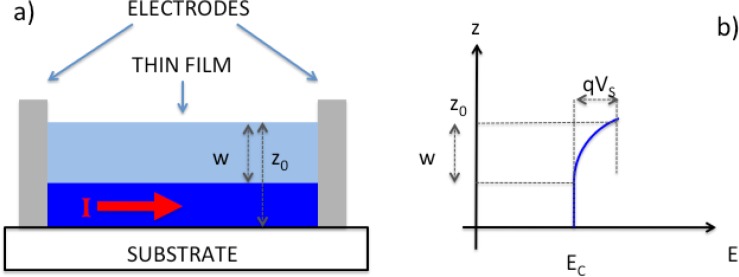
Schematic representation of the structure (**a**) and working principle (**b**) of thin film gas sensors.

The thin film approach exploits a compact layer with thickness as close as possible to the space charge layer width (W). In the abrupt approximation, the macroscopic conductance of this structure is described by a non-conducting layer of thickness W, located at the outermost surface, and a conducting layer below it, having thickness z_0_-W. A schematic representation is provided in Figure 1. It is evident from this picture that sensitivity is optimized by reducing the film thickness at values close to W, so that the SCL extends through the whole film.

The advantage of the thin film approach is the reproducibility, thanks to its simple geometry that does not involve percolative paths for electrical conduction (which occur in thick films). On the other hand, it features a limited surface area, close to the geometrical area, enhanced by a relatively small factor depending on the surface roughness. This limits the sensitivity of the device with respect to performance obtained with thick-film technology.

**Figure 2.**
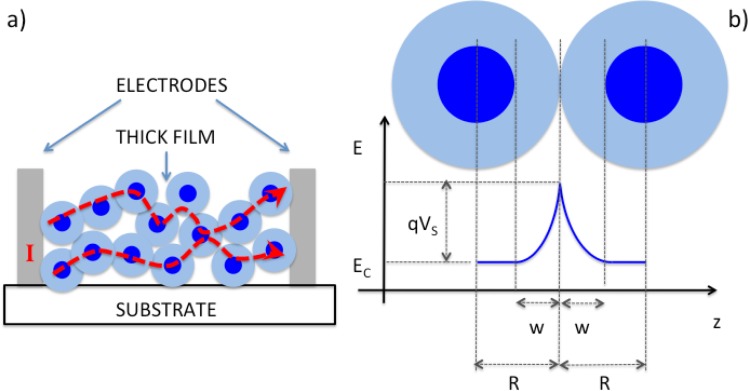
Schematic representation of the structure (**a**) and working principle (**b**) of thick film gas sensors.

The thick film approach exploits a granular and porous structure, allowing gas diffusion within the layer so that the inner portion of the layer is also exposed to gaseous molecules. A schematic representation of the thick film structure and band-bending across grains is shown in Figure 2. In this case, sensitivity is optimized by reducing the grain size to the SCL width.

With respect to the thin-film situation, the thick film structure allows one to exploit the whole volume of the layer (by properly controlling the porosity and the grain size) and the surface area is much enhanced, leading to an increased response amplitude to gases. On the other hand, the conduction mechanism is more complicated and occurs through percolative paths connecting neighboring grains [5]. This makes the capability to prepare layers composed by grains with uniform size critical, since small deviations in the grain size distribution and network from sample to sample will lead to dramatic differences among nominally identical samples [5].

Besides these two ideal models, several approaches have been developed trying to find the optimal compromise between the two structures. It is worth noting that this sensing mechanism is highly sensitive to a broad range of chemicals, but is weakly selective. Different strategies have been developed to address selectivity with semiconducting metal oxide chemiresistors, the most common concern the use of sensor arrays and the use of temperature protocols. The former is based on a sensor array, composed by sensors with different specific sensitivity, and a pattern recognition software handling the response of the whole array [6,7]. The latter exploits the effect of the sensor temperature on the gas-oxide interaction. Properly modulating the sensor temperature by means of temperature pulses or *ad-hoc* designed temperature profiles, the sensor resistance variation with time becomes highly sensitive to the kinetics of interaction with gases, thus allowing one to extrapolate features needed for selectivity from a reduced number of sensors, even a single sensor, depending on the application [8,9].

So far, the need is both for highly sensitive sensors but also to develop sensors with different sensitivities. In the next paragraphs, solutions developed at the SENSOR Lab with respect to these needs are reviewed, describing the synthesis process of materials, their functional performances and the integration of the different materials obtained in a sensor array to develop an electronic nose suited for different applications.

## 3. Gas Sensors Devices

### 3.1. Thin Film Based Devices

As mentioned in Section 2, a drawback of thin film technology is the development of layers featuring a limited surface area. The research activity of the SENSOR Lab was strongly focused on this topic during the 90s, leading to the development of the RGTO (rheotaxial growth and thermal oxidation) technique [10,11]. It is based on sputtering, a thin film technique [12], but it is suited to produce a monolayer of oxidized droplets featuring a high surface to volume ratio.

The RGTO technique consists of two deposition steps: first the preparation of a metallic thin film by DC magnetron sputtering from a metallic target on a substrate kept at a temperature higher than the melting point of the metal, then the thermal oxidation cycle in order to get a metal oxide layer with stable stoichiometry.

The deposition of the metal on a substrate at a temperature higher than the melting point causes the formation of spherical clusters instead of flat films. During the oxidation treatment, the metallic layer is first annealed in air at a temperature lower than its melting point. This slightly oxidizes the metallic droplets without fusing them in a continuous and compact layer. The slight oxidation strongly increases the melting temperature of the material that is further annealed in air at higher temperature. In this way, the material reaches a stable stoichiometry without losing the droplet-like morphology, which features high surface to volume ratio, suitable for gas sensing application.

The sensor device is then completed by depositing Pt interdigitated electrodes over the oxide layer, while a Pt meander, acting both as heater and temperature probe is deposited on the rear side of the substrate.

**Figure 3.**
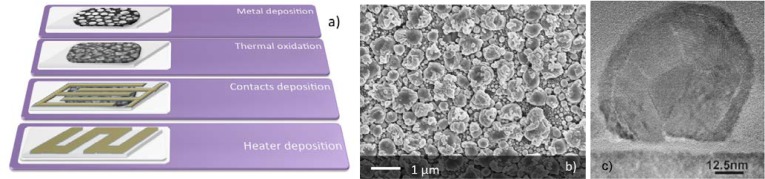
Different steps for the preparation of chemical sensors based on RGTO thin-films (**a**); SEM image of the morphology of a SnO_2_ layer prepared by the RGTO technique (**b**). TEM image of single particle of the RGTO layer (**c**), (reprinted from [13] with permission of Elsevier, copyright 2000).

The basic steps for preparation of RGTO sensors are summarized in Figure 3(a). Figure 3(b) shows the morphology of the RGTO layer at the microscale, while the details of a single particle (solid droplet) composing the layer is reported in Figure 3(c). Such a TEM image shows that each droplet is further composed by crystallites with size of the order of 10 nm [13].

The versatility of the sputtering method has also been used to develop different materials by means of the RGTO technique, such as SnO_2_ and In_2_O_3_[11]. With the aim of addressing specific sensitivity toward different compounds and developing a set of sensors suited for integration in an electronic nose system, RGTO thin-films catalyzed with metallic nanoparticles, as well as multilayer structures featuring the presence of interfaces of different oxides, such as, for example, the SnO_2_-Fe_2_O_3_ system, featuring high sensitivity to O_3_, have been developed [14–16].

Despite the versatility of sputtering, the RGTO technique can only be used to prepare oxides of metals having a relatively low melting point. Tungsten oxide is one of the oxides that can’t be prepared through this method, but, at the same time, it is a widely used material for gas sensing applications, especially for its high sensitivity to oxidizing gases such as NO_2_ and O_3_[17].

**Figure 4.**
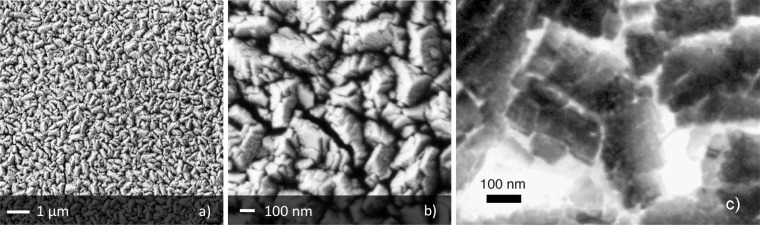
SEM images of the WO_3_ layer prepared by the thermal evaporation method developed at sensor lab, (**a**) and (**b**). TEM bright field image of the structure of WO_3_ agglomerate (**c**) (reprinted from [18] with permission of Elsevier, copyright 2005).

To overcome the trouble of the reduced surface are of WO_3_ layers prepared by sputtering method, we further developed a method based on thermal evaporation [18]. It consists on the direct sublimation of a metallic tungsten wire by Joule effect in an oxygen rich atmosphere. The application of a constant voltage to the W-metallic wire allows to heat it to a high temperature (over 2,000 °C) and thus to induce a fast sublimation of the material. The substrate is kept at a short distance from the source (15 mm) and hold at a much lower temperature (600 °C). This freezes the tungsten oxide material arriving over the substrate surface and inhibits the grain coarsening phenomenon. The oxide stoichiometry is further stabilized by means of an annealing treatment in air. As shown in Figure 4(a,b), the synthesized layer features a porous and homogeneous morphology on the large scale, while at the micro- and nano-scale it is composed by agglomerates with size in the μm range, further structured in nanosized grains.

The TEM image reported in Figure 4(c) further shows the structure of the agglomerates, revealing that the size of single crystallites is between 50 and 100 nm. Further details on the synthesis parameters and structural properties can be found in [18]. Sensors prepared by this method revealed about five times more sensitivity than their sputtering counterparts [18].

### 3.2. Nanowires Based Devices

Metal oxides in the form of nanowires are interesting for their peculiar morphology and their exceptional crystalline features, the first assuring a high surface to volume ratio necessary to maximize surface related properties such as the ones governing chemical sensing transduction principles, while the latter guarantees stable crystallinity and therefore electrical properties over long-term operation, *i.e.*, a required quality for an industrial application of any kind of sensor in real environments.

After the first method proposed for the preparation of metal oxide in the form of nanobelts [19], plenty of literature has been devoted to different experimental techniques that may lead to the formation of these quasi one dimensional structures. First of all there are two main groups that can be distinguished: top down and bottom up technologies. The first starts from bulk structures, reducing them to nanometric dimensions usually with lithographic techniques, while the latter involve a direct assembly in the desired morphology that can be obtained from vapor or liquid phase. At the beginning the research was focused on the vapour phase methods that were producing, with cheap instrumentation, high quality nanostructures in terms of crystallinity and stoichiometry.

We have thoroughly studied the deposition using evaporation and condensation from powder in controlled environments using a different experimental set up. Tin oxide was preferred over other oxides thanks to its well known chemical sensing properties and the easy preparation conditions, but indium and zinc oxide were also studied [20–25].

The experimental procedure consists in the evaporation of the powder (metal or metal oxide) at high temperatures in a controlled atmosphere at pressures lower than hundreds of millibar and the following mass transport of the vapour towards the substrates kept at lower temperatures with respect to the source evaporation region. Argon, an inert gas, is used for the mass transport in order to avoid unwanted reactions with the oxide vapour. Critical variables are the distance between the source and the substrates and the temperature gradient that are controlling the supersaturation conditions in the vapour phase causing the condensation in the desired morphology.

In order to increase and finely control the production of nanowires we have preferred a catalyst assisted deposition. The catalyst particles were deposited on alumina substrates by sputtering using a shadow mask to control the deposition area and ensure an uncontaminated part for pad and electrical contact deposition to assure a good mechanical adhesion.

**Figure 5.**
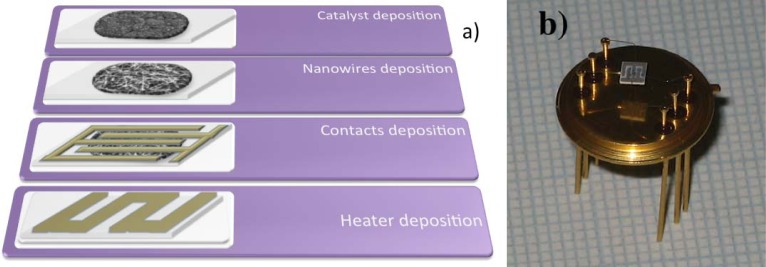
Different steps for the preparation of nanowires based chemical sensors (**a**); sensor prototype (**b**).

**Figure 6.**
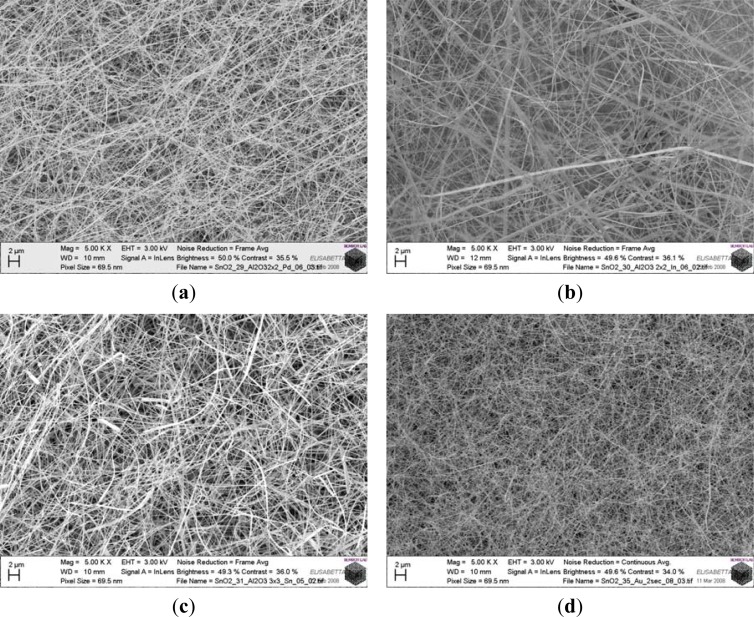
Different morphologies of tin oxide nanowires deposited with (**a**) palladium, (**b**) indium, (**c**) tin and (**d**) gold catalyst at 400 °C approximately.

The layout of the transducer and the deposition steps are illustrated in Figure 5. The morphology of the prepared nanostructures depends on the growth conditions. Concerning tin oxide the morphologies obtained depends on the selected catalyst and the different deposition temperatures. The use of Pt catalyst assures the formation of nanowires in a wide range of temperatures from 350 to 500 °C, considerably larger than the one obtained using other catalysts. The use of tin and palladium as catalysts lead to the formation of wires with micron lateral size or tridimensional structures. Figure 6 reports different nanowires morphologies that can be obtained with tin oxides together with different catalyst to assist the growth.

### 3.3. Functional Characterization

To compare the performance of the developed materials and sensors, we chose the detection of chemical warfare agents (CWAs), a challenging application, where high sensitivity, selectivity and fast response time are required.

In particular we worked with CWA simulants compatible with laboratory tests [26], using acetonitrile (ACN) as a simulant for cyanide compounds and dimethyl methylphosphonate (DMMP) as simulant for the nerve agent Sarin.

In this field, the threshold concentration is usually defined by means of the Immediately Dangerous for Life and Health (IDLH) value. It means that a person exposed to such a concentration has 30 min to leave the contaminated area before experiencing permanent effects [26]. The IDLH value for the nerve agent Sarin is about 0.05 ppm [27], while it is a few tens of ppm for cyanide compounds [28].

**Figure 7.**
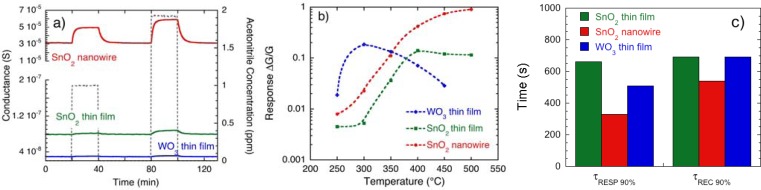
Response of sensors based on SnO_2_ RGTO thin films, SnO_2_ nanowires and WO_3_ thin film to acetonitrile (a simulant for cyanide compounds): (**a**) dynamic responses measured at the sensor temperature of 450 °C; (**b**) temperature effects on response amplitude to 2 ppm of acetonitrile; (**c**) response and recovery times of sensors to 2 ppm of acetonitrile measured at the working temperature of 450 °C.

Figure 7(a) shows the dynamic response of the three basic materials (SnO_2_ RGTO thin film, WO_3_ thin film prepared by the method based on thermal evaporation detailed in Section 3.1 and SnO_2_ nanowire) toward acetonitrile (ACN) at the sensor temperature of 450 °C. Figure 7(b) shows the temperature effect on response amplitude of these three sensors to 2 ppm of ACN.

First of all, it can be noted in Figure 7(a) that the nanowire sensor is much more conductive with respect to thin film devices, the former showing a baseline conductance of the order of 300 μS, compared with the baseline of 0.025 μS and 0.08 μS of the WO_3_ and SnO_2_ thin films, respectively. This is a consequence of the different structure and network of the nanowire and thin film devices. Thin films show a granular structure (see Figures 3(b) and 4), with a large number of grain boundaries that decrease the macroscopic conductance of the layer. Differently, as shown in Figure 6, the nanowire device is composed by long crystallites with wire morphology, which behave as highly conductive channels, thanks to their high degree of crystallinity [29]. Thus, the electrons’ conductive path from one electrode to another will be largely carried out along the nanowire bodies, encountering a few nanowire-nanowire interfaces and resulting in a higher macroscopic conductance.

Concerning the response amplitude, the nanowire sensor is the best performing, showing a response to ACN that is about one order of magnitude higher than the responses of thin films. Furthermore, the nanowire sensor reaches its maximum response at the temperature of 500 °C, much higher than the optimal temperature of the WO_3_ thin film (300 °C) and SnO_2_ thin film (400 °C). This is an advantage because the higher temperature fastens sensors’ response and recovery dynamic, furthermore it reduces poisoning effects that can be induced by exposure to aggressive gases such as nerve agents [27].

A comparison of response and recovery kinetics shows that both nanowires and thin films show quite similar values (at the same temperature). For example, response times to ACN, defined as the time required by the sensor conductance to reach the 90% of its final value, are about 5–10 minutes for the three sensor materials, while recovery times to ACN, defined as the time required by the sensor conductance to restore the baseline value within 90%, are about 10–13 minutes (Figure 7(c)). Response and recovery kinetics of chemiresistors arise from the combination of different phenomena, including gas diffusion through the sensing layer and chemical reactions between gaseous molecules impinging over the oxide surface and reactive sites available over the surface. Our senor layout (see Section 3.1 for details) features the deposition of electrodes over the surface of the sensitive layer. This choice has been specifically done to maximize the importance of the outermost layers of sensing material with respect to the inner ones. Despite the fact the whole current will flow through the whole volume of the device, a larger portion will pass through the surface layers (directly exposed to gases), which provide a more straightforward connection between electrodes with respect to inner layers (whose gas exposure is affected by diffusion phenomena). Thanks to this layout, the optimization of parameters such as porosity and film thickness becomes less critical (at least concerning diffusion phenomena) than the situation encountered working with a layout featuring electrodes located under the sensing layer [30]. For example, despite the fact the WO_3_ layer shown in Figure 4 has a thickness of about 300 nm and a certain degree of porosity, its structure is much less open than the structure of a nanowire layer, and a much slower diffusion is expected, compared to nanowires. Concerning the RGTO thin film, its particular structure, composed by almost a monolayer of interconnected solid droplets, strongly reduces the diffusion process making the whole surface of the sensing element quickly available to gaseous molecules. Despite these differences, the kinetics of the three layers do not feature appreciable differences, suggesting that the sensor layout is effective in reducing diffusion effects.

On the other hand, all three sensor materials show higher recovery times than response times. This is a common feature in metal oxides and is usually ascribed to thermally activated chemical reactions occurring at the oxide surface [4,31].

**Figure 8.**
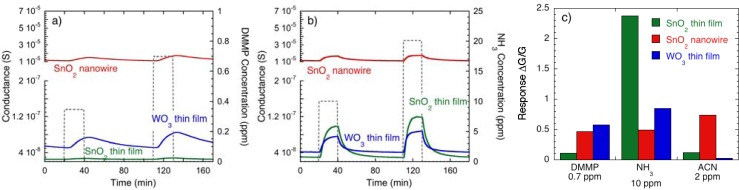
Dynamic response of sensors based on SnO_2_ RGTO thin films, SnO_2_ nanowires and WO_3_ thin film to (**a**) DMMP (simulant for Sarin nerve agent) and (**b**) ammonia. (**c**) Comparison between the response of sensors based on SnO_2_ RGTO thin films, SnO_2_ nanowires and WO_3_ thin film to 0.7 ppm of DMMP, 10 ppm of NH_3_ and 2 ppm of ACN.

Another important feature is selectivity, which is obtained working with a sensor array composed by sensors showing different specific sensitivity. As an example, the dynamic response of the three target sensors chosen in this work toward DMMP and ammonia are shown in Figure 8(a,b), respectively. In these plots, the conductance scale is settled as in Figure 7(a), where the response to ACN is shown, in order to allow a comparison between the response amplitude to the three gases. These plots clearly show the different sensitivities of the three sensors: the SnO_2_ nanowire sensor exhibits a specific sensitivity to ACN, with a high response already at concentrations lower than the IDLH value of corresponding real CWAs (cyanide compounds), while its response to ammonia and DMMP is much lower. The SnO_2_ thin film shows a specific sensitivity to ammonia, whose response is much higher than the response observed to ACN and DMMP. Finally, the WO_3_ thin film shows specific sensitivity to DMMP, with much lower responses to ACN and ammonia.

Despite the fact that the different sensitivity of the WO_3_ layer can be ascribed to the different material (WO_3_*vs.* SnO_2_), it’s interesting to note that the SnO_2_ RGTO thin film and the SnO_2_ nanowires shows the same cassiterite tetragonal SnO_2_ phase [10,21]. These differences were ascribed to the different structure of the SnO_2_ surface: nanowires show surfaces corresponding to well-defined crystalline planes, while polycrystalline layers show rounded surfaces, composed by more crystalline planes. These features can promote different adsorption processes depending on the properties of the surface exposed to gases, such as, for example, the diverse degree of crystallinity [4].

Concerning stability, both the morphology and structure of tin dioxide nanowires are stable at temperatures even much higher than the operating temperature of the sensing devices, and stability tests have been performed at 800 °C and atmospheric pressure. Nanowires are also extremely resistant to mechanical stress as reported in [32]. The preparation method used at SENSOR (see Section 3.2) permits one to grow nanowires directly over the sensor substrate (depositing in a second step the contacts and heating element). Since the development of the sensor device does not require nanowire removal, the nanowires remain strongly bonded to the substrate, thanks to the chemical bonding occurring during the synthesis process. This ensures the robustness of the sensor structure.

Furthermore, it has been reported that nanowire based gas sensors show an enhanced long-time stability, with respect to sensors based on nanoparticles, ascribed to the single crystalline structure of nanowires, which reduces the sintering phenomena affecting films with nanoparticle morphology [33,34].

Overall, these features indicate that nanowires can be successfully used in electronic nose systems [35–37]. In particular, the observed different sensitivities suggest that thin film and nanowire technology can be used together to develop electronic noses featuring enhanced selectivity [37]. This was analyzed in detail by means of Principal Component Analysis (PCA) in [37], showing that an electronic nose composed by both thin film and nanowire sensors was more sensitive and more selective than an electronic nose realized by means of any one technology.

## 4. From Chemical Sensors to Electronic Noses: Prototypes and Real Scenario Applications

MOX sensor arrays have been successfully integrated in Artificial Olfactory Systems (AOSs), most often known as Electronic Noses (ENs). In particular, SENSOR developed some prototypes, one of which was the ENs EOS835 (a detailed description can be found in [38]) whose applications in real scenarios will be presented. The system is now produced for the market by SACMI scarl (Imola, Italy), to which the know-how on sensor preparation and integration in olfactory machines has been transferred.

**Figure 9.**
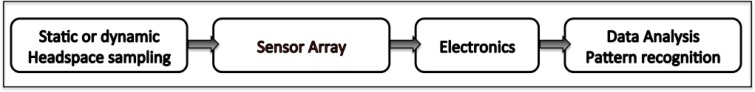
Schematic of the four main building blocks of an EN.

An EN, whose building block schematic is shown in Figure 9, is constituted by a unit dedicated to headspace (HS) sampling, a sensor array, the electronics managing the system and a pattern recognition part. HS sampling can be performed in either static or dynamic ways, the former being more reproducible while the latter is selected in case of need for increased sensibility, due to the possibility of extracting larger sample volumes, while paying attention to the possible changes in selectivity deriving from the concomitant extraction .of compounds with low volatility.

During the years the MOX sensor arrays synthesized by SENSOR have found applications in many different fields, such as odor chemical agent warfare detection, [37] food quality evaluation [38,39], process monitoring [29,40] and microbial contamination diagnosis [41,42].

Among the latest applications in which the EN EOS835 has been tested, the field of food quality and safety control is particularly relevant. ENs can indeed be exploited for quality evaluation, freshness and shelf-life determination, microbial contamination diagnosis, identification of errors occurred during the processing of raw products, *etc*. A review on the application of ENs in the field can be found in [43]. The capability of ENs in comparing an *in-standard* with an *out-of-standard* product is mainly related to the changes appearing on the qualitative and/or quantitative composition of the volatile chemicals present in the sample headspace. This point is easily understood when thinking that mammals have used their sense of smell for centuries to identify what could be eaten from what is not to be consumed. During the last years, the SENSOR Laboratory has been working on both *in vivo* and *in vitro* cases as for microbial contamination of vegetable and fruit derived food, while studies have also been conducted on adulteration of high quality products with lower quality analogous products and on the possibility of identifying line errors in the process line. Hereafter, some relevant case studies are presented.

### 4.1. Diagnosis of *Alicyclobacillus* spp. in Soft Drinks and Fruit Juices

*Alicyclobacillus* spp. (ACB) are spore forming, Gram positive, non-pathogenic, thermoacidophilic bacteria having the surprising capability of surviving the pasteurization processes undertaken in the process line to reduce microbial loading. Among their secondary metabolites, developed some days after bottling, there are 2,6-alophenols that impart unpleasant smells and tastes to the final products. Since 1982, ACB are causing relevant economic damages to producer companies that sell contaminated products without knowing this. Since the contamination can happen at any point of the process line, at present it is impossible to envisage effective strategies to avoid the presence of ACB, especially in vegetable- and fruit-derived foodstuffs. Another critical point is related to analytical techniques: currently, contamination diagnosis is performed by detecting the chemical secondary metabolites (especially guaiacol, recognized as the main off-flavor), which means identifying its presence once the product is already spoilt [44]. EN EOS835 was applied to soft drinks [45] and fruit juices [45] in order to verify the possibility to perform an early diagnosis of the presence of ACB.

**Figure 10.**
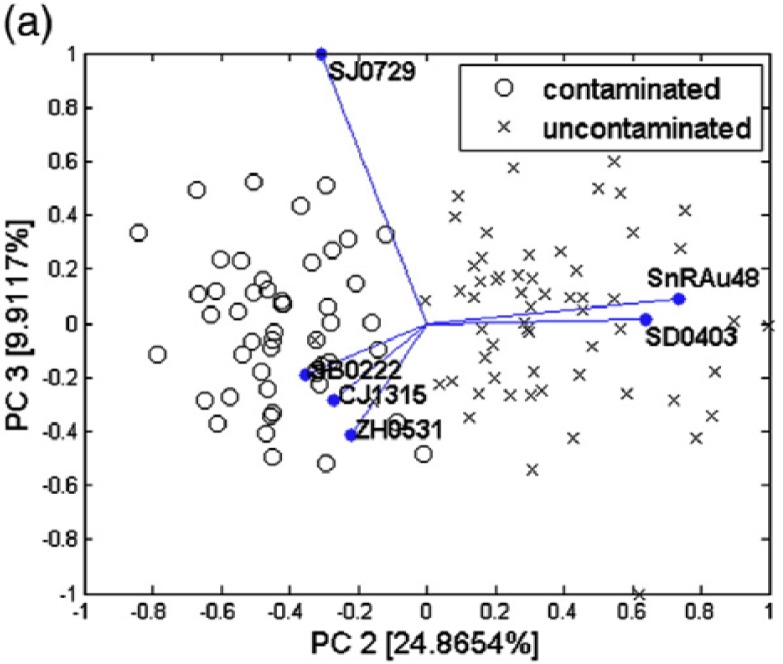

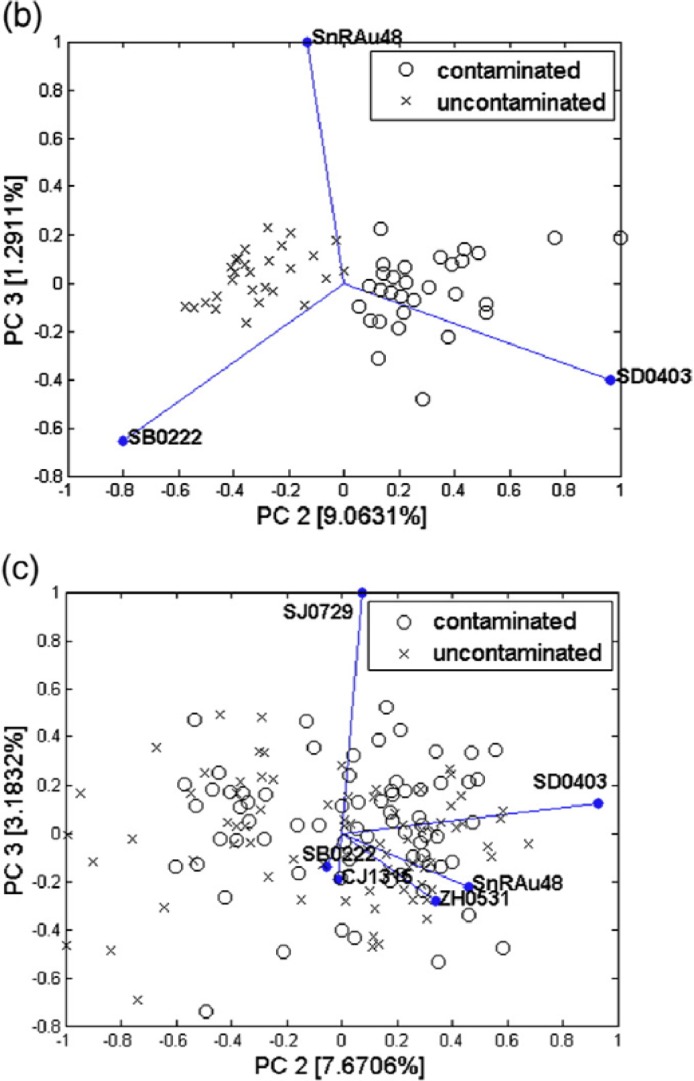
Biplots showing the capability of EN EOS835 in discriminating uncontaminated and ACB contaminated samples of soft drinks ((**a**) peach-based; (**b**) pear-based and (**c**) apple based). Reprinted from [45] with permission of Elsevier Food Research International, Copyright 2012.

**Figure 11.**
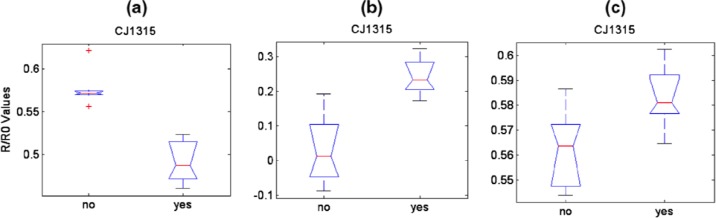

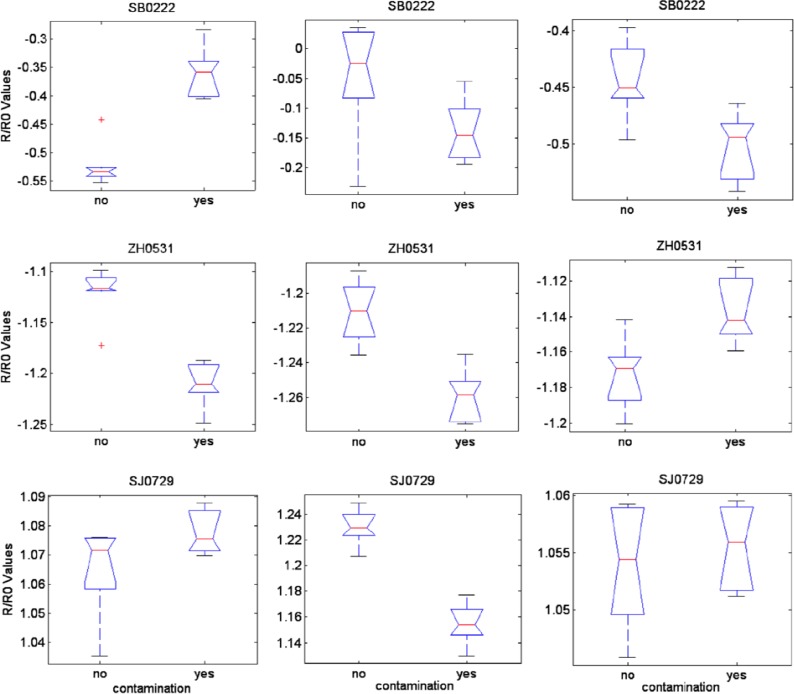
Box plots of sensors able to identify ACB contaminated (“yes”) and uncontaminated (“no”) juice samples. Feature considered is R/R_0_. Columns: (**a**) peach; (**b**) orange; (**c**) apple. Reprinted from [46] with permission from Elsevier, Copyright (2012).

**Figure 12.**
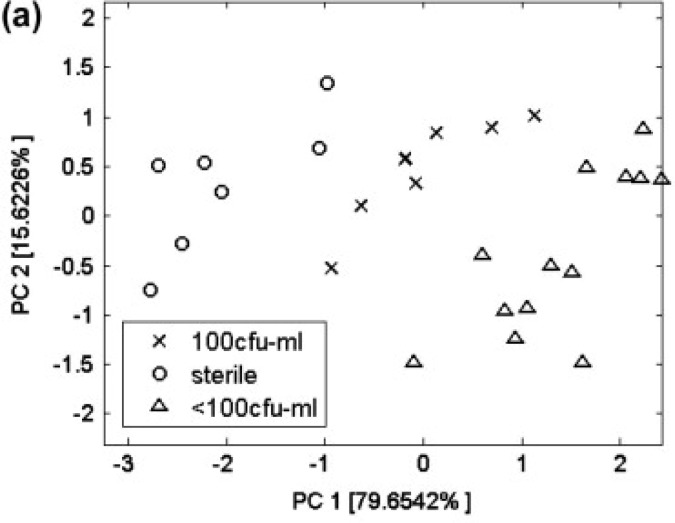

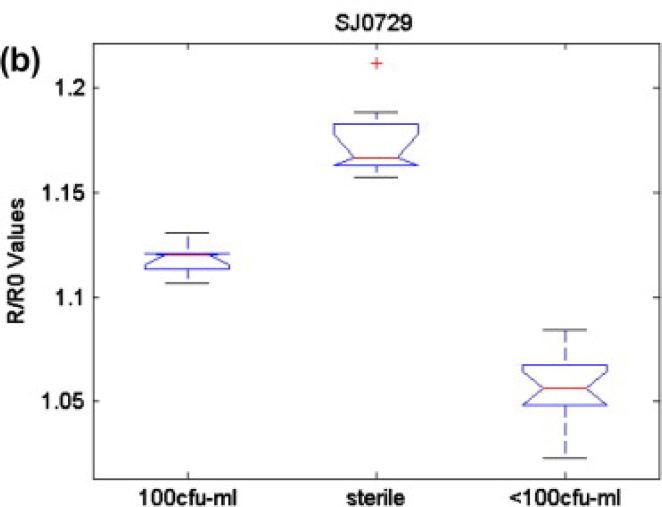
EN sensitivity for different cfu of ACB in fruit juice. (**a**) PCA score plot of data related to orange juice inoculated with 100 and <100 cfu/mL of *A. acidoterrestris*; (**b**) Box plot of the best performing sensor of the array for the same measurements. Reprinted from [46] with permission from Elsevier, Copyright 2012.

The EN was revealed to be able to identify contaminated products at very low contamination levels for naturally contaminated soft drinks (see Figure 10). As for fruit juices, after an artificial inoculation, EN could separate data related to uncontaminated and contaminated juices with a remarkable sensitivity (Figures 11 and 12) as compared with that shown by *classical* analytical techniques (both chemical and microbiological). An important parameter to verify in view of possible industrial applications of ENs is indeed their sensitivity. Usually, we state that chemical analytical techniques and microbiological assays have the advantage of being more sensitive than artificial olfactory systems. However, Gobbi *et al.*[46] found a sensitivity of 10 and <102 cfu/mL for peach and orange juice, respectively, while Concina *et al.*[45] identified the ACB presence at a level of tens of copies/mL by polymerase chain reaction analysis. It is however worth mentioning that the skill of EN in recognizing contaminated juices/soft drinks was found in both cases to be dependent on the matrix: whereas for orange-, peach- and pear-based beverages the artificial olfactory system showed a remarkable analytical capability, apple-based drinks made the sensor array almost blind, strongly reducing the diagnostic capability if not suppressing it completely. This effect was found despite the contamination levels, as well as sample treatment before analysis, both comparable with those of the other matrices.

Similarly, the discrimination capability was dependent on the active layer considered: not all the sensors in the array were able to separate contaminated from uncontaminated samples.

In the frame of the study on soft drinks an informal panel test (constituted by colleagues and students) was also carried out in order to simulate the behaviour of a consumer not specifically trained in analyzing foods and beverages (see Table 1). A complete correspondence between the judgments given by the panelists and the EN was found, again confirming the ability of these machines in simulating mammalian olfaction, that is another relevant skill of AOSs.

### 4.2. Identification of Fraud in a High Quality Food Product: The Case of Extra Virgin Olive Oil

Another relevant field of application of AOSs concerns the possibility of identifying fraudulent practices in high quality foodstuffs. An interesting case is related to the dilution of extra virgin olive oil (EVOO) with low quality hazelnut oil. This fraud has unfortunately become rather frequent in the Mediterranean basin over the years and poses problems of two orders: the first one is related to the consumers’ rights (and to some health risks due to allergies related to hazelnuts), while the second one is more of socio-economical relevance. EVOO is indeed mainly produced in low income regions of Europe and it can represent the most relevant source of sustenance for farmers, who are heavily damaged by the fraudulent adulteration with other vegetable oils. Discrimination between EVOO and hazelnut-diluted EVOO (HD-EVOO) is very difficult with classical techniques, due to a very similar fatty acids content composition.

**Figure 13.**
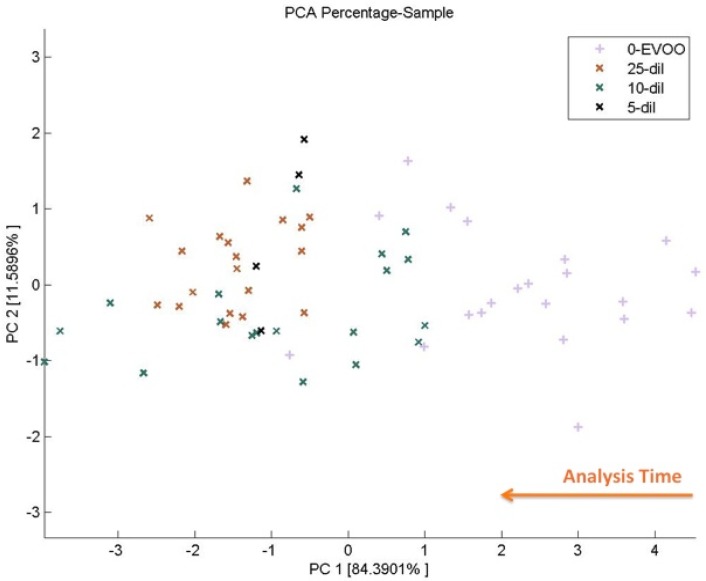
PCA score plot of data related to pure EVOO and HD-EVOO samples (5 to 25% V/V). Reprinted from [47] with permission from AIP, Copyright 2012).

In this context, EVOO was diluted with increasing percentages of hazelnut oil (5 to 25% V/V) and the skill of EN EOS835 in recognizing adulterated samples was then tested [47]. As shown in Figure 13, data referred to pure EVOO was well separated on PCA plane, while data referring to different HD-EVOO samples, although discriminated from the previous ones, lie unclustered on the plane.

In this case, we have to mention that EVOO is a food matrix changing rather fast over time. These natural changes, typical in every foodstuffs, are of particular relevance for EVOO, which undergoes, for instance, (photo)oxidation processes altering the global composition of the headspace. EN is sensitive to these changes, which reflect in a dispersion of data on the PC1 axis. Care has then to be taken when dealing with this kind of product: a continuous monitoring on both the matrix and the sensor array has to be exerted to keep each relevant analytical parameter under control.

### 4.3. Identification of Errors in the Process Line

Another relevant field of application for ENs is related to the identification of errors occurring in the process line that can spoil the sensory quality of the final product. A typical example is represented by an over-heating of the raw material generating an off-flavour known as *cooked smell* for processed tomatoes. Tomato-based products are widely consumed throughout the World, both raw and cooked, and are used as ingredients in many dishes, sauces and drinks. They serve as major, inexpensive low-fat food sources providing energy, high-quality protein, fiber, vitamins, pigments, as well as other nutrients, and moreover their consumption seems associated with a lower risk of certain types of cancers, in particular of the prostate, lung and stomach [48]. The FAO statistical database (FAOSTAT) indicates a production of billion tonnes of raw fruit in 2009, being China, USA and Italy the major producers (with ∼34, 14 and 6 million of tonnes, respectively).

**Figure 14.**
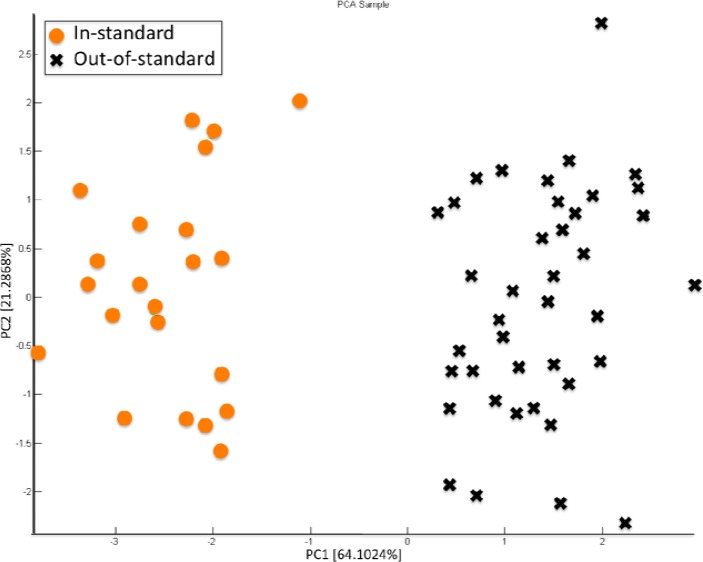
PCA score plot of data related to *in standard* (orange circles) and *out-of-standard* (black x) processed tomatoes. Reprinted from [47] with permission from AIP, copyright 2012.

The characteristic tomato and tomato-derived product flavor is determined by volatile substances. These substances develop partly during ripening, and partly during the comminution of the ripe fruit, as an effect of the enzymes activated. About 400 volatiles have been identified, among which those characterizing fresh tomato flavor are mainly derived from fatty acids and amino acids. During the processing of the raw material, chemicals responsible for the flavor undergo both qualitative and quantitative changes. Moreover, a final pasteurisation step is also carried out in order to lower the microbial load, which can result in additional changes of the flavor of the final product. These changes can result in either an improving or worsening of the global sensory properties. Heat treatments are critical both in extent and in duration: final content in sugar, amino acids and acids is indeed determined in these steps. Volatiles partly evaporate and new components are formed, which are responsible for the “cooked” smell of final products [49]. Currently, the identification of this off-flavour is made by a trained human panel, smelling certain lots and discarding those evaluated as not suitable for sale according to sensory quality. The EN EOS835 was employed to investigate the possibility of identifying lots of tomato sauce affected by the *cooked smell* and thus discarding them from production [47]. With this aim, an oversterilization step was simulated by heating 1 min at 120 °C in autoclave. Figure 14 reports the PCA data analysis of sensorily suitable tomato pulp and oversterilized product. Data related to the two classes are completely separated on the PC1 axis, thus demonstrating the capability of the EN EOS835 in recognizing the *cooked smell*.

The reported examples highlight the potential of the artificial olfactory systems as reliable candidates for food quality and safety monitoring in industrial quality control laboratories.It should be noted that the sensor technology allows for a rapid and simple screening of the products: in most cases, indeed, no sample treatment prior to analysis is required, sample throughput is remarkable and, after adequate training, the instrument can work in stand-alone mode.

As a final remark, it is worth reminding the reader that sensor technology still presents some limits, related for instance to sensor drift, sensitivity to humidity, and sensor reproducibility [50]. These limits can represent an obstacle to a definitive admission of ENs in industrial quality control labs. However, there is currently a noteworthy effort, both scientific and technological, to overcome the mentioned issues, through both software corrections (for instance in case of sensor drift) and technical improvements (for instance by applying a humidity correction directly inside the machines). We are thus waiting for improved machines, more reliable and robust, to come and find widespread industrial applications.

## 5. Conclusions

In this paper we’ve reviewed the research activities on gas sensors carried out at the SENSOR Lab in Brescia (Italy), detailing the needs and the conceptual steps that led to the development of the rheotaxial growth and thermal oxidation (RGTO) technique in the 90s, aimed at enhancing the surface area in layers prepared through thin-film technologies, and, later on, to the development of thermal evaporation methoda to address the same goal with WO_3_ (which can’t be prepared by RGTO). Finally, the research to develop innovative materials featuring high sensitivity and stability led to the exploitation of nanowire technology to prepare gas sensors. The different technologies developed at SENSOR are then compared with respect to the detection of chemical warfare agents, showing that one technology is not more sensitive than the other in a general way, differently, each material features its own specific sensitivity. So far, it is not proposed to use the newer technology to replace the older, but rather to integrate them in a electronic nose system to enhance selectivity.

Finally, the performances of the electronic nose (EN) developed at SENSOR are shown in three food-quality control applications. In particular, it is shown that the electronic nose can already successfully detect contamination of beverages from *Alicyclobacillus spp* at early stages, with higher sensitivity than other existing analytical techniques. The EN was revealed as a suitable tool to identify frauds, such as for example, addition of hazelnut oil to extra-virgin olive oil—which represent a challenging problem for analytical techniques due to the similar composition of the two oils (as for fatty acids content). In addition, the EN was revealed able to detect overcooking errors in the production line of tomato sauces.

## Figures and Tables

**Table 1.** Results of informal panel test carried out on soft drinks uncontaminated and contaminated by ACB. Beverage numbers correspond to: 1 peach juice-based, 2 pear juice-based, 3 apple juice-based. Reprinted from [45] with permission of Elsevier Food Research International, copyright 2012.

	**Beverage No.**	**Panellist ID**	**1**	**2**	**3**	**4**	**5**	**6**
SMELL	Guaiacol 1 ppm		Minty, medicinal	Hospital	Hospital	Minty, hospital	No response	Herbal
	Guaiacol 4 ppb		Smoky	No response	Hospital, medicinal	Slightly minty	No response	Hospital
	1 - NC		Floreal	Fruity	Floreal, fruity	Fruity	Fruity	Fruity
	1 - C		Hospital	Fruity	Fruity	Fruity	Unagreeable	Green apple
	2 - NC		Fruity	Floreal	Floreal	Herbal	Fruity	Floreal
	2 - C		Fruity (peach), floreal	Herbal	Fruity (peach), hospital	Fruity	Fruity, minty	Herbal
	3 - C		Green apple	Apple	Floreal	Herbal	Fruity	Medicinal
	3 - NC		Fruity	Floreal	Floreal	Fruity	Herbal	Fruity

TASTE	1 - NC		Floreal	Fruity	Floreal	Fruity	Fruity	Fruity
	1 - C		Hospital	Fruity	Fruity	Fruity	Unagreeable	Green apple
	2 - NC		Floreal	Fruity	Floreal	Fruity	Fruity	Fruity
	2 - C		Floreal	Herbal	Fruity, medicinale	Fruity	Fruity, minty	Herbal
	3 - NC		Apple	Floreal	Floreal	Fruity	Herbal	Fruity
	3 - C		Green apple	Apple	Fruity, floreal	Herbal	Herbal	Medicinal

§ Abbreviations: NC - not contaminated, C - contaminated.
